# Assessing the Status of Wild Felids in a Highly-Disturbed Commercial Forest Reserve in Borneo and the Implications for Camera Trap Survey Design

**DOI:** 10.1371/journal.pone.0077598

**Published:** 2013-11-04

**Authors:** Oliver R. Wearn, J. Marcus Rowcliffe, Chris Carbone, Henry Bernard, Robert M. Ewers

**Affiliations:** 1 Department of Life Sciences, Imperial College London, Ascot, United Kingdom; 2 Institute of Zoology, Zoological Society of London, London, United Kingdom; 3 Institute for Tropical Biology and Conservation, Universiti Malaysia Sabah, Kota Kinabalu, Sabah, Malaysia; University of Tasmania, Australia

## Abstract

The proliferation of camera-trapping studies has led to a spate of extensions in the known distributions of many wild cat species, not least in Borneo. However, we still do not have a clear picture of the spatial patterns of felid abundance in Southeast Asia, particularly with respect to the large areas of highly-disturbed habitat. An important obstacle to increasing the usefulness of camera trap data is the widespread practice of setting cameras at non-random locations. Non-random deployment interacts with non-random space-use by animals, causing biases in our inferences about relative abundance from detection frequencies alone. This may be a particular problem if surveys do not adequately sample the full range of habitat features present in a study region. Using camera-trapping records and incidental sightings from the Kalabakan Forest Reserve, Sabah, Malaysian Borneo, we aimed to assess the relative abundance of felid species in highly-disturbed forest, as well as investigate felid space-use and the potential for biases resulting from non-random sampling. Although the area has been intensively logged over three decades, it was found to still retain the full complement of Bornean felids, including the bay cat *Pardofelis badia*, a poorly known Bornean endemic. Camera-trapping using strictly random locations detected four of the five Bornean felid species and revealed inter- and intra-specific differences in space-use. We compare our results with an extensive dataset of >1,200 felid records from previous camera-trapping studies and show that the relative abundance of the bay cat, in particular, may have previously been underestimated due to the use of non-random survey locations. Further surveys for this species using random locations will be crucial in determining its conservation status. We advocate the more wide-spread use of random survey locations in future camera-trapping surveys in order to increase the robustness and generality of inferences that can be made.

## Introduction

With rates of forest loss and degradation in Southeast Asia exceeding all other tropical regions [1], and the majority of remaining forest existing in a highly disturbed state [2], [3], there is now an urgent need for accurate assessments of the impacts on wildlife in the region. This situation applies especially to Borneo and to the five species of felid inhabiting the island: Sunda clouded leopard *Neofelis diardi* (Vulnerable on the IUCN Red List), leopard cat *Prionailurus bengalensis* (Least Concern), flat-headed cat *Prionailurus planiceps* (Endangered), marbled cat *Pardofelis marmorata* (Vulnerable) and bay cat *Pardofelis badia* (Endangered). For all of these species, we still have a paucity of information on their distributions, population statuses and responses to land-use changes. This is particularly the case for the bay cat, a Bornean endemic which has been called “the world's least known felid” [4]. Certainly, very few confirmed records of it exist [5] and it has variously been suggested to be either tolerant [6], [7] or intolerant of habitat disturbance [8].

A number of targeted field studies of Borneo's terrestrial fauna have recently been undertaken [9]–[11], with some focussing on wild felids [5], [12]–[14]. Importantly, there has been a rapid increase over the last decade in the use of camera traps for conducting such studies [15], allowing intensive surveys to be made over larger areas with reduced effort in the field. This has led to significant extensions in the known distributions and habitat tolerances of many species [16]–[19], including Borneo's wild cat species [12], [20], [21]. However, it remains the case that few camera trap surveys have been done beyond the boundaries of protected areas, in forests which are not pristine and not sustainably managed (but see [9], [22], [23]). Less than 6% of land area in Indonesia and Malaysia is protected (IUCN categories I–IV, [24]) and most landscapes are now dominated by highly-disturbed forests which have undergone multiple rounds of logging [3], [25], [26]. It is only these highly-disturbed forests that still occur over sufficiently large and contiguous areas to potentially conserve viable populations of felid species occurring at very low densities, such as the clouded leopard [14], [27], [28].

The proliferation of camera-trap studies has allowed more robust inference on the relative abundance of highly cryptic species than has been possible before. This has led to a re-assessment of the supposed rarity of some taxa, including the Asiatic golden cat *Pardofelis temminckii* [29]–[31]. The bay cat, on the other hand, has remained consistently rare in camera-trap surveys throughout its range, usually appearing at least one order of magnitude less frequently than other Bornean felids [7]. Since detection frequencies are a function of both abundance and detection probability [32], the rarity of bay cat records could reflect low detection probability rather than low population densities. Low detection probability in camera trap surveys can result from a range of factors, broadly categorised as factors that reduce camera sensitivity, and factors that reduce the chances of animals encountering cameras. An important species-specific correlate of camera-sensitivity is body size [33]. However, the bay cat is comparable in size to the other three small cats of Borneo: the leopard cat, flat-headed cat and marbled cat [4]. In terrestrial surveys, reduced detection probabilities are obviously expected for arboreal species, but it also does not seem likely that the bay cat is more arboreal than the other cat species: all direct sightings have been made on or very close to the ground [5] and its morphology is consistent with terrestriality [6].

Low detection probabilities can also result from avoidance of the particular habitat features on which camera-trapping surveys typically focus. Ever since their early use in mark-recapture studies [34], camera traps have been deployed preferentially where the presence of a focal species is deemed most likely – usually on trails, roads, water points or mineral licks – in order to increase individual capture probability. It is now common and accepted practice to use these non-random deployment locations in general wildlife surveys and then calculate an index of relative abundance [32]. In some cases, researchers have stated that cameras were deployed “randomly” but actually refer to a two-step process in which potential deployment zones (typically squares of a grid overlain on the study area) are selected at random and then cameras are deployed non-randomly within these zones. Given that deployment zones are typically much larger than the area actually sampled by a camera trap –2 km^2^ grid squares are often used (e.g. [35]) compared to sensors with maximum detection zones mostly less than 2×10^−4^ km^2^ [36] – species may be detected less frequently, or not at all, if they avoid certain habitat features within the focal area.

Choosing ‘optimal’ locations for deploying cameras in this way violates a key assumption of sampling theory – the random selection of sample units – and necessarily limits the scope of inference of a study to the specific conditions found at the survey locations. Inferences made beyond this limited subset of features of a habitat or landscape are likely to be biased, even though this is routinely done. To our knowledge, no camera-trapping study conducted in Borneo, or indeed more broadly in the Palaeotropics, has used strictly random locations (within 5–10 m of a pre-marked point, e.g. [37]). This may have implications for the currently inferred abundance and understanding of habitat use for all species, including felids. Owing to the prevailing use of non-random camera trap surveys, up to now it has not been possible to explicitly test for these possibilities.

We aimed to assess the status of wild felids in a highly-disturbed commercial forest reserve, gathering together both incidental sightings and camera-trapping records from strictly random locations. Given that we used random camera locations, we also investigated the potential for non-random survey designs to interact with non-random space-use by animals, which would cause biased inferences about relative abundance. To do this, we investigated felid space-use patterns with respect to anthropogenic habitat features, which have typically been the focus of camera trap surveys, and also compared our relative abundance estimates to those from previous camera-trapping studies conducted in the region. As a result, we suggest that prevailing camera trap methods have indeed confounded assessments of felid species rarity. Our findings have implications for the conservation of our focal species, as well as the study design of camera-trap surveys in general.

## Methods

### Study Area

This study was carried out in Kalabakan Forest Reserve (4° 33′ N, 117° 16′ E) in the state of Sabah, Malaysia, and forms part of the Stability of Altered Forest Ecosystems (SAFE) Project [38]. Kalabakan Forest Reserve lies within the Yayasan Sabah Forest Management Area and, as such, has been subject to multiple, intense rounds of logging, beginning in 1978 and ongoing until the early 2000 s. This has led to a heterogeneous landscape composed of stands which have undergone varying intensities and timings of log extraction, using both tractor-based and high-lead yarding methods. During the logging, a network of regenerating skid trails, logging roads and log-landing areas was also created (approximately 10% of land area [39]). As a result, there is a range of habitat types currently exhibited in the reserve, from grassy open areas and low scrub vegetation, to lightly logged forest on steep slopes and in rocky areas, but the timber volume remaining in the area is mostly very low (below 10 m^3^ ha^−1^). In addition to the logged forest areas, large portions of the reserve have been terraced and planted with oil palm, or have been salvage logged in preparation (removing all trees above 25 cm diameter at breast height). Medium-resolution (250 m) land cover maps for 2010 [26] therefore indicate that just 54% of the area of the Kalabakan Forest Reserve (2,240 km^2^) still retains natural forest cover.

### Data Collection

We deployed remotely-operated digital cameras (Reconyx HC500, Holmen, Wisconsin, USA) in the north-east of the Kalabakan Forest Reserve (4° 42′ N, 117° 34′ E), overlapping with the SAFE Project experimental area (72 km^2^). We had full permission from the land-owners and concession holders, Yayasan Sabah and Benta Wawasan Sdn Bhd, to conduct this study in the reserve. We also had an access license in place from the Sabah Biodiversity Council for the use of camera traps at the study site. Our study was approved by the Zoological Society of London Animal Ethics Committee and the work detailed here did not involve any direct sampling methods or the collection of any specimens.

**Figure 1:**
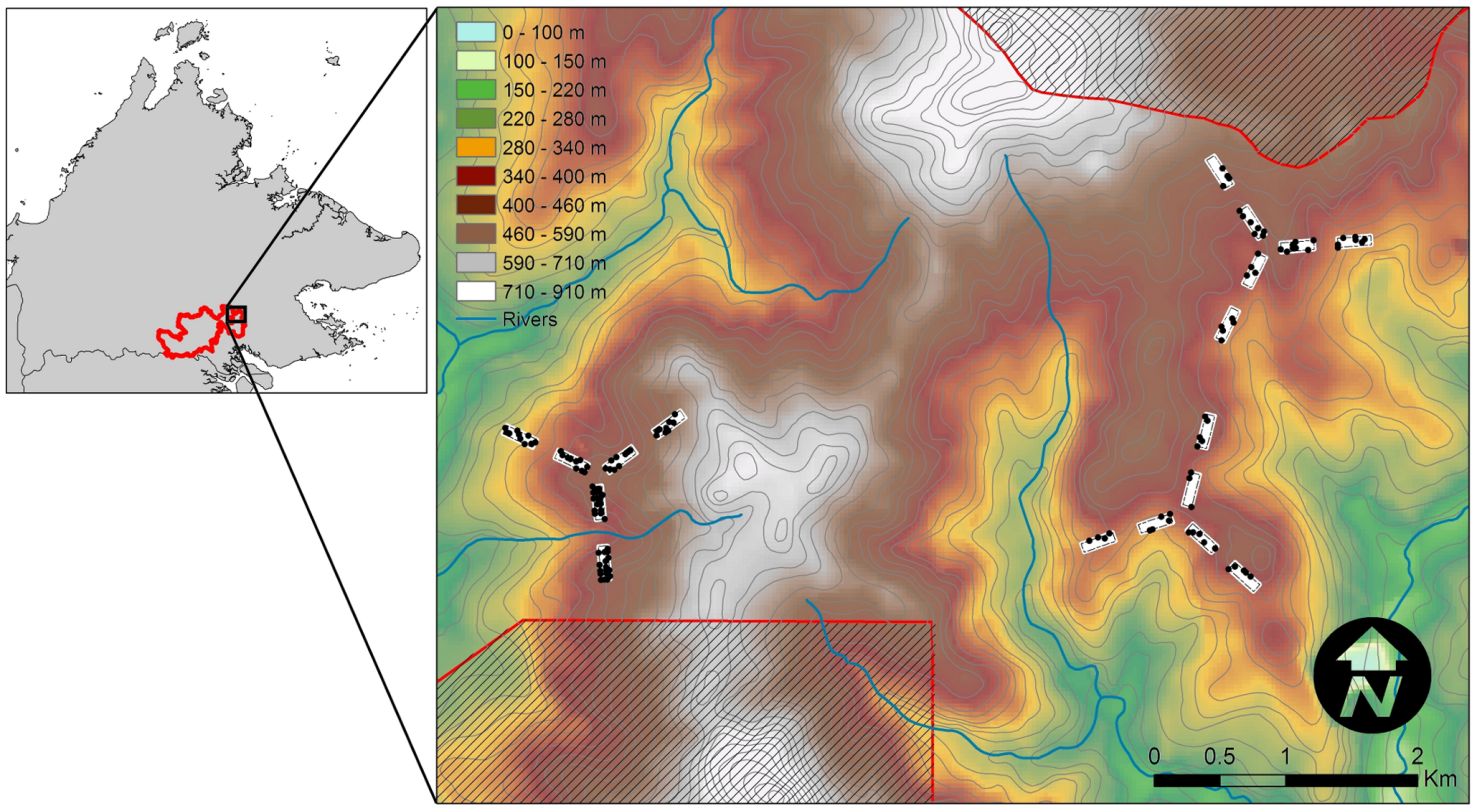
Locations sampled using camera traps within the Kalabakan Forest Reserve, Malaysian Borneo. Camera traps were deployed at random locations (black points) within 1.75 ha plots (white rectangles), clustered into three groups placed deliberately to control for elevational effects. Shaded areas lie outside the Kalabakan Forest Reserve and are composed of the Brantian-Tatulit Virgin Jungle Reserve (to the south) and the Ulu Segama Forest Reserve (to the north). Inset shows the location of Kalabakan Forest Reserve (red outline) within the Malaysian state of Sabah, northern Borneo.

We sampled 135 locations between May and December 2011 for an average of 49 camera-trap nights (CTNs), giving a total effort of 6650 CTNs. Camera traps were deployed inside 18 separate plots, each covering 1.75 ha, which were clustered into three groups (Fig. 1). This design was chosen to overlap with the sampling locations of the SAFE Project [38]. The SAFE Project has attempted to control the confounding effects of elevation by stratifying the study site and only sampling within a relatively narrow range centred at ∼450 m; the elevation of our plots reflects this stratification. For each plot, we established a 4×12 grid of points (23 m spacing) in the field using a tape measure and GPS receiver (Garmin GPSMAP 60CSx, Olathe, Kansas, USA). There is a margin of error associated with these methods, but we ensured that field teams marked the grids with no consideration of the practicalities of whether or where a camera might be set at each grid point. Grid points can therefore be considered to be truly random within plots. Cameras were deployed at a random subset of points within each grid (mean  = 8 points per grid), as close to the marked points as possible. Necessarily, large obstructions in the camera's detection zone (such as rock boulders or large tree buttresses) were avoided, but cameras were always deployed strictly within 5 m of marked points. We usually set cameras at a height of 30 cm, to maximise detection for a range of species, but some cameras were set higher (and faced downwards), depending on the situation found at each random location. No bait or lure was used and disturbance to vegetation was kept to a minimum. Cameras were programmed to take 10 consecutive photographs for each trigger event (over approximately 5 seconds). We noted if the detection zone contained a logging road (wide, heavily compacted ground, sometimes with gravel remnants, no canopy cover), a skid trail (width of a tractor, canopy cover, recruiting vegetation at ground-level, earth-banked sides), footpath or none of these (which we term “off-trail”).

In parallel to the camera-trapping effort, we also recorded the location and time of all incidental records of felids obtained during the course of the fieldwork detailed here and across all of the research activities at the SAFE Project. These data are inherently biased towards less-cryptic, large-bodied and diurnal species, and sampling was highly non-random in space and time. We excluded periods for which consistent reporting from the SAFE Project was unavailable, leaving approximately 10 months of observations between August 2010 and August 2011.

We also conducted an extensive literature search for previous camera-trapping studies done on any of the five Bornean felid species. We used the ISI Web of Science (www.isiknowledge.com, using various searches on the vernacular and scientific species names, as well as “camera trap*” and the names of Southeast Asian countries) to locate published and peer-reviewed studies, and supplemented this with other published and unpublished sources we were aware of or which were cited in other sources. For inclusion in our database, studies had to report the total number of CTNs conducted, as well as the number of independent captures. Where data were not presented in a suitable form, we attempted to contact authors directly for clarification.

### Data Analysis

Image sequences were judged to be independent capture events if they a) contained different individuals or b) were separated by more than an arbitrary 1 hour. We present a detection frequency (*d*) – often referred to in the literature as a relative abundance index – for each species, which is the number of independent captures per 100 CTNs (accounting for camera failure).

We modelled the binary detection or non-detection of each species as a function of the habitat features at camera locations, using a generalised linear model with binomial errors and a logit link function. Factor-level simplification was done using chi-squared likelihood ratio tests. For the clouded leopard, we also tested for an interaction between habitat features and the sex of individuals. We used Fisher's exact test with the null hypothesis that the number of detections of males and females is not conditional on whether a camera is placed on a logging feature (road or skid trail) or not.

Using empirical data from our literature survey of camera-trap records, we constructed a probability density function for the expected detection frequency of each of the five cat species. This was done by taking bootstrap samples of the data (n = 10,000) and calculating the overall detection frequency each time. We stratified samples by study site, to give each study site equal weight and to ensure that each observation within randomisations was independent.

We then used the bootstrapped median detection frequencies to estimate the minimum survey efforts required to detect each species with a given probability, incorporating the uncertainty in the distributions of *d* using the 95% quantile values. Detections of a species *D* were modelled as a Poisson process, with a rate parameter λ (detections per camera-trap night). For consistency with the camera-trapping literature we used the detection frequency *d*, which has units per 100 CTNs, i.e. λ = *d*/100, and for a survey conducted over *n* camera trap nights, the expected number of detections E(*D*)  =  *λn* = *nd*/100. Given Poisson distributed detections, we can use the cumulative exponential distribution to calculate the probability *p* of obtaining at least one detection of a species after surveying for a given number of camera trap nights. Using *d* instead of *λ* gives
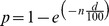



Plotting this for a range of *n* gives a detectability curve [40]. We can determine the number of camera trap nights required for a given cumulative probability or “confidence” either graphically or by solving for *n*. For example, for 90% confidence (*p* = 0.9) in detecting a species, the minimum sampling effort required is calculated by
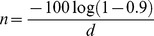



All analyses were done in R version 2.12.2 [41].

## Results

Camera-trapping yielded 504 photos of wild cats, consisting of 41 independent captures across 29 locations (21% of random locations sampled). It took 873 camera trap nights to detect four out of the five Bornean felid species. The clouded leopard was detected most frequently and at the most locations, followed by the leopard cat (Table 1). Both of these species were also detected by incidental sightings. The two rarest felids from camera-trapping were the marbled cat and bay cat; the marbled cat was detected more times than the bay cat but at fewer locations (Table 1). Neither of these species were observed during incidental sightings. In contrast, the flat-headed cat was not detected during camera-trapping but was directly sighted. This observation consisted of a single individual crossing a narrow logging road (∼5 m wide and bordered with ∼2 m of dense scrub and *Coelorachis glandulosa* grass) approximately 70 m from the nearest stream (∼5 m width) and at 180 m elevation.

**Table 1:** Wild felid species recorded from the Kalabakan Forest Reserve, Sabah, Malaysia.

Common name, scientific name	Direct sightings	Camera trapping			
		No. photos	Independent captures	Detection frequency (*d*)	Naive occupancy
Sunda clouded leopard *Neofelis diardi*	1	267	14	0.211	0.081
Marbled cat *Pardofelis marmorata*	0	89	9	0.135	0.052
Bay cat *Pardofelis badia*	0	64	8	0.120	0.059
Leopard cat *Prionailurus bengalensis*	13	84	10	0.150	0.067
Flat-headed cat *Prionailurus planiceps*	1	0	0	0	0

Direct sightings are incidental records obtained during the course of fieldwork (August 2010 to August 2011). Independent captures from camera trap image sequences are of different individuals or images obtained more than 1 hour apart. Detection frequency *d* is the number of captures per 100 camera trap nights. Naive occupancy is the proportion of sampled locations at which the species was detected.

The minimum adequate models for site detection probabilities of each species revealed a significantly higher probability of detection on logging features (z = 2.639, p = 0.008), i.e. logging roads and skid trails, for clouded leopard and on skid trails only for marbled cat (z = 2.615, p = 0.009). The probabilities of detection on the back-transformed scale were 0.195 (SE  = 0.066) and 0.040 (SE  = 0.020) for clouded leopard at locations on and off logging features, respectively, and 0.200 (SE  = 0.103) and 0.025 (SE  = 0.014) for marbled cat on and off skid trails, respectively. There was an indication of sex-specific differences in habitat feature use in clouded leopard (p = 0.061): male clouded leopards were only detected on logging features, whilst two-thirds of detections of females were made off-trail. No habitat feature variables were retained in the minimum adequate models for the leopard cat and bay cat, which means that the null hypothesis of random use of habitat features was not rejected. Note, however, that 7 of 8 independent captures of bay cat were off-trail.

We were able to obtain useable data on previous detections of the five Bornean felid species from 34 separate camera trap studies across at least 27 study sites. This represents a combined effort of approximately 62 years of fieldwork between 1998 and 2011, resulting in 1,212 felid detections over 142,672 CTNs. The amount of effort spent surveying for each species was unequal, being largely dependent on the geographic range of the species: bay cat survey effort (60,914 CTNs) has been approximately half that of marbled cat (120,231 CTNs). Without exception, these studies used non-random survey locations.

**Figure 2:**
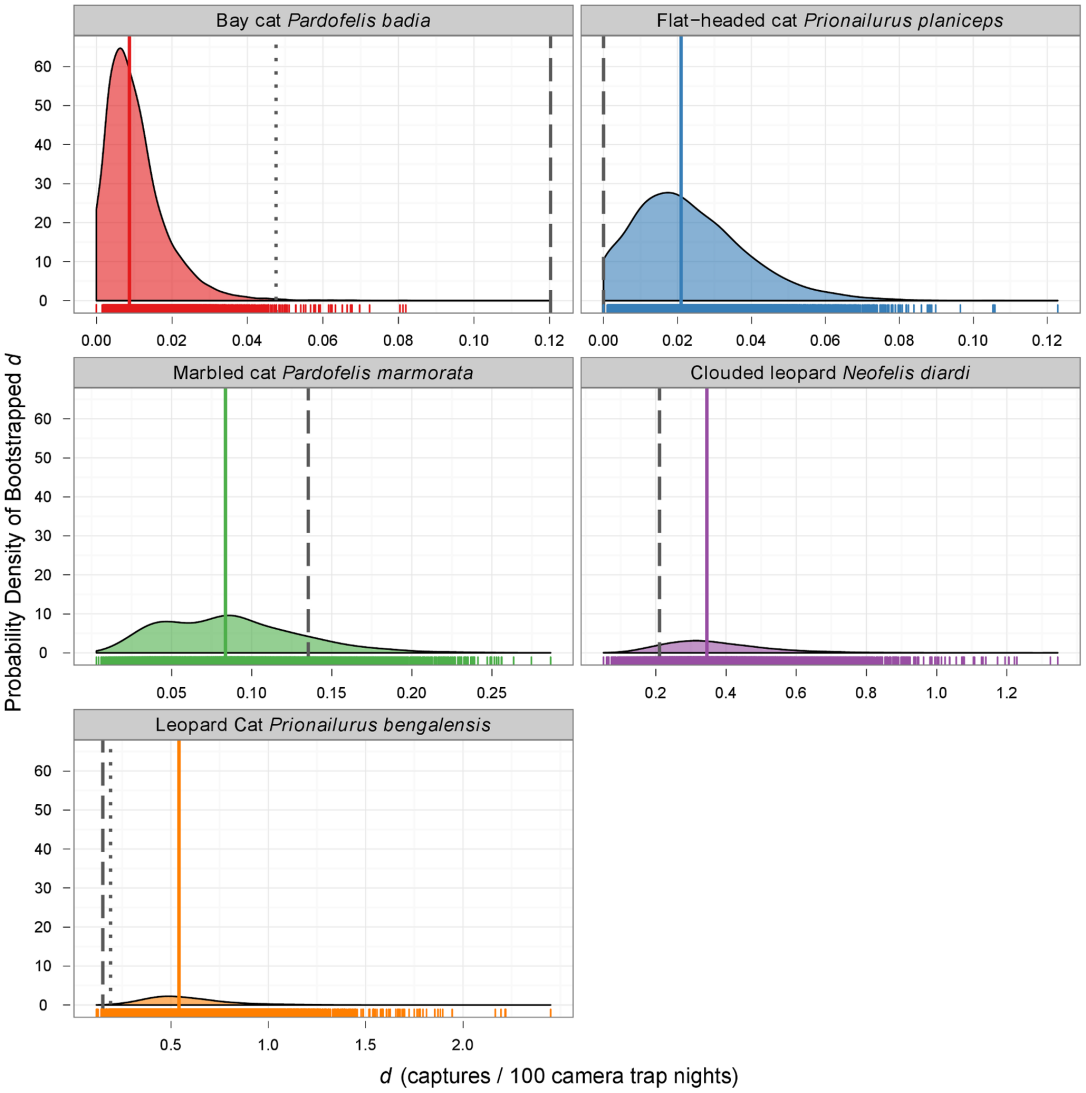
Probability density functions for bootstrapped values of detection frequency (*d*) derived from previous camera-trapping studies. Data for each of Borneo's felid species were obtained from 34 studies conducted between 1998 and 2011 and bootstrap randomisations (n = 10,000) were stratified according to study site. Each panel shows the probability density function obtained by kernel density estimation, the median *d* from bootstrap samples (solid line) and *d* obtained in the current study, using strictly random survey locations (dashed line). Dotted lines for bay cat and leopard cat show *d* calculated after excluding off-trail survey locations. Note that the x-axis is not consistent across panels.

We calculated the detection frequency (*d*) across all studies combined for each species. These showed an order of magnitude difference between the relatively commonly detected leopard cat (*d* = 0.701) and clouded leopard (*d* = 0.320) to the more rarely detected marbled cat (*d* = 0.079), flat-headed cat (*d* = 0.021) and bay cat (*d* = 0.015). Once we accounted for the unbalanced and autocorrelated nature of the dataset using a stratified bootstrap sampling approach, the expected detection frequencies were lower in the case of the bay cat and leopard cat, but the rank order of detection frequencies amongst the species was the same (Fig. 2). The detection frequencies observed in the current study lay within the 2.5% and 97.5% quantiles of the bootstrapped distribution for the clouded leopard, marbled cat and flat-headed cat, but were significantly higher and lower than expected for the bay cat and leopard cat, respectively (Fig. 2). The detection frequency we obtained for bay cat from random survey locations was more than 10 times larger than that expected from previous studies.

Owing to this significant difference for the bay cat and leopard cat, we decided *post hoc* to compare our overall detection frequencies for these species with those we would have obtained at our study site with a traditional trail-based survey, by excluding data obtained from off-trail cameras. We found the same qualitative differences between random and non-random camera placement designs within our study as we had found between our results and those found in the camera-trapping literature: detection frequencies were 2.5 times larger and 0.8 times smaller for the bay cat and leopard cat, respectively, for a survey design with off-trail cameras than one without (dotted lines, Fig. 2).

**Figure 3:**
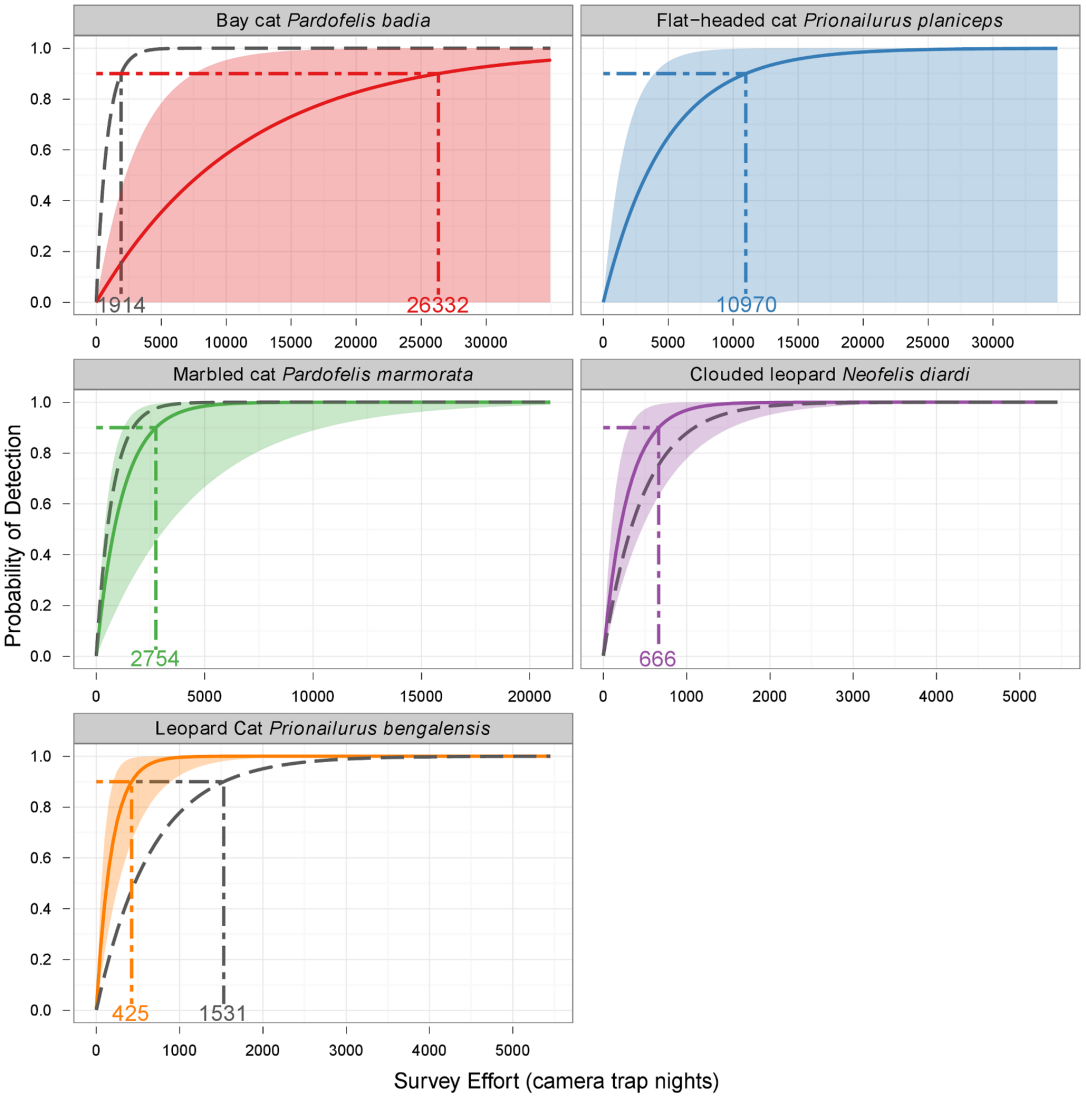
Detectability curves and minimum required survey efforts calculated using a Poisson model for detections. Detectability curves for each of Borneo's felid species were plotted using Eq. 1 and minimum survey efforts calculated using Eq. 2 with a “confidence” of 90% and per-trial probability of success estimated using *d* (captures per 100 camera trap nights). Solid lines use median *d* from bootstrap samples of camera trap data obtained from previous studies (with shading corresponding to the 95% quantiles of *d* from bootstrap samples) and dashed lines use *d* obtained in the current study using random survey locations (except for flat-headed cat, which was not detected by camera-trapping in our study). For each detectability curve, survey efforts required for 90% confidence are indicated with dot-dash lines and annotated on the axes. Note that the x-axis is not consistent across panels.

We calculated the minimum survey efforts required for each species based on the data from previous studies (Fig. 2) and Eq. 2 (using *p* = 0.9). Huge disparities between species were revealed, ranging from more than 26,000 CTNs required for bay cat to just 425 CTNs for leopard cat (Fig. 3). We also calculated the worst-case scenario (using the 2.5% quantile for *d*) and this extended the survey effort required substantially in all cases (Fig. 3): the requirement more than doubled for clouded leopard and leopard cat and more than trebled for marbled cat. For the bay cat and flat-headed cat, the lower bound did not exclude zero, so we could not rule out the possibility of never detecting these species regardless of survey effort. For the bay cat, we compared the required effort suggested by previous studies to that suggested by the current study using random locations: the required effort was reduced by more than 24,000 CTNs for the detection frequency observed in our study (Fig. 3). This was also associated with a comparatively small increase of 1,106 CTNs in the minimum effort required for the leopard cat.

## Discussion

Using camera traps and direct sightings, we confirmed the presence of all five Bornean wild felids in the Kalabakan Forest Reserve. Moreover, the four species detected by camera-trapping were estimated to have a relative abundance of the same, or higher, order of magnitude as previous studies conducted elsewhere (Fig. 2). We also investigated possible biases in the relative abundances derived from past camera-trapping efforts, caused by the non-random survey designs which have typically been used. Importantly, we found both inter- and (for clouded leopard) intra-specific differences in the use of habitat features. In addition, there were significant differences between the relative abundances we obtained using random camera locations and those from previous studies, and we found similar differences in comparing on- and off-trail locations within our own survey design. We suggest these differences are evidence of biases, caused by an interaction between patterns of animal space-use and the non-random deployment of camera traps at locations chosen by researchers.

Many book and journal pages have been devoted to exploring issues of survey design for monitoring and assessment of populations [32], [42]–[44] and we do not wish to recapitulate all of the design principles that have been recommended. However, random selection of sample units is central to most sampling schemes [45]. Given our findings, it is clear that this should also be central to the design of camera trap surveys. We have shown that this allows small-scale habitat-use to be investigated, and provides a stronger basis for inferences about relative abundance. There are, however, some important instances where a non-random design might be preferred for species monitoring, such as when the detectability of individuals can be explicitly modelled using mark-recapture methods. Although such models require high capture, and indeed recapture, rates and employ stricter assumptions [46], they allow inferences about absolute abundance for the limited subset of species which can be individually identified from camera trap images. Occupancy methods, too, account for detectability (of a species) and, although having similarly demanding data requirements (a large number of independent sample locations may be required for anything other than common species), may also provide a strong basis for inference about the status of a population, if not abundance *per se* [47]. Beyond monitoring, non-random designs might also be considered in rapid, preliminary surveys which seek only to determine if a species is present in an area, rather than its population status.

We found all five species of Bornean felid in the Kalabakan Forest Reserve. We are aware of only three other sites which have confirmed records of all five species: Deramakot Forest Reserve [12], Danum Valley Conservation Area [48] and Tabin Wildlife Reserve [20]. These sites range from pristine (Danum), through to sustainably managed (Deramakot) and selectively logged until the late 1980s (Tabin). The addition to this list of the Kalabakan Forest Reserve, a highly-disturbed commercial forest reserve which has undergone decades of sustained logging until very recently, therefore extends this list to the full range of forest disturbances present in Borneo.

Taken together, our results suggest that the large areas of highly-disturbed natural forest in the region could play a greater role in the conservation of wild felids than is currently recognised. It does still remain to be known if populations of these five species would be viable in disturbed forest in the long-term and we therefore echo previous assertions of the importance of undisturbed forest [49], [50]. However, we did obtain photographic evidence of breeding within our highly-disturbed study site for clouded leopard (one female with cub) and calculated a relative abundance that was similar to those from previous studies, mostly done in more intact sites (Fig. 2). The habitat tolerances of the bay cat are poorly known, but our results using random survey locations indicate that the relative abundance of this species may be of the same order of magnitude as the other wild felid species in disturbed habitats.

We did not detect the flat-headed cat in the period of our camera-trapping survey. Given a total survey effort of 6650 CTNs, and based on the detectability of this species in previous studies (Fig. 3), we had a 25% chance of failing to detect this species. Most records of this species have been obtained within 3 km of large bodies of freshwater (including rivers and lakes) and below 100 m elevation [21]. None of our random camera trap locations were near large water bodies and, due to a stratification inherent in the survey design, locations were at a mean elevation of 432 m (range: 278–543 m). If our survey design had been random with respect to elevation, then it is possible that we would have also detected this species with our camera traps.

We found significantly higher probabilities of detection along logging features and skid trails for clouded leopard and marbled cat, respectively. In contrast, the leopard cat and bay cat were not found to preferentially use logging features and apparently exhibited random use of habitat features. Habitat-use patterns have rarely been investigated using camera-trapping data, due to the ubiquity of non-random sampling and the narrow range of habitats this necessarily focuses upon. As a result, studies have generally focussed on modelling detection rates as a function of the properties of the trail or road itself [51]–[53]. The only other study that we are aware of that has used strictly random locations found marked differences between on- and off-trail trapping rates for a range of species on Barro Colorado Island, Panama, including a six-times higher trapping rate on trails for ocelot, *Leopardus pardalis* [37]. Our own results support this for two other species of wild felid.

We also found evidence of sex-specific differences in the use of habitat-features, with female clouded leopards avoiding logging-related features, possibly due to the risk of aggression or infanticide on the part of males [13]. Heterogeneity in capture probabilities between the sexes has been previously noted in clouded leopards [13], [27] and is an important source of bias in parameter estimation under a mark-recapture framework [54]. Our results suggest that females may be more likely to be recaptured, and heterogeneity reduced, if traditional trail-based survey locations are supplemented with off-trail locations. For the marbled cat, the finding that detection probabilities were eight-fold higher on skid trails relative to other features including logging roads should be a point of further investigation, and may help to explain the low detection frequencies of this species in previous studies (Fig. 2).

We obtained data from previous camera-trapping surveys carried out across Southeast Asia to characterise for the first time the probability distribution of *d*, the detection frequency, for each species of felid, and from this provide general recommendations for minimum survey efforts. Though rarely available, this is vital information for the effective design of wildlife surveys. The detection frequencies observed for our random survey locations deviated significantly from the expectation based on previous studies in the case of the bay cat and leopard cat. Together with the differences observed between off-trail and on-trail locations within our own study, this suggests that non-random sampling regimes have resulted in biased inferences with respect to the relative abundance of these species, especially for the bay cat.

The bay cat was listed as Endangered when it was last assessed under the IUCN Red List categories and criteria [55]. This was on the basis of an estimated population size of less than 2500 mature individuals and a projected population decrease of more than 20% over 12 years. Since this assessment was made, the proliferation of camera trap studies has yielded a number of new records for the bay cat, both published (this study, [12], [20], [56], [57]) and unpublished [58]–[60], which has greatly expanded the known habitat tolerances of this species in terms of both disturbance and maximum altitude (up to ∼1500 m). It now seems likely that the bay cat can occur in highly-disturbed forest, as well as the vast areas of upland forest (300 to 1,000 m elevation) and possibly even montane forest (>1,000 m elevation) in the proposed Heart of Borneo transboundary conservation area [61]. Our finding that bay cat detection frequencies increase substantially using random camera locations could also indicate a widespread underestimation of its relative abundance. Considering these facts, a case could be made for reconsideration of the conservation status of the bay cat during the next IUCN Red List cat assessments, due to be completed by 2015. However, important uncertainties still remain in assessing the future for the bay cat, especially with regards to land-use trends in the Yayasan Sabah Forest Management Area [25], which is emerging as an apparent stronghold for the species, but also more broadly in the ongoing land-use planning process for the Heart of Borneo area [62].

Camera traps are typically placed non-randomly in order to obtain a greater quantity of data per unit of effort expended or money spent. We have shown here that, for certain species such as the bay cat, this may not always be appropriate. Cameras and other wildlife sensors, such as sound recorders, are rapidly improving in terms of sensitivity, battery life, data storage capabilities and robustness to adverse environmental conditions, and are therefore producing more data per unit of effort or monetary input than ever before. As a result, the traditional barrier to strictly random survey locations – the paucity of data that may result – is rapidly being overcome. There will always be a role for non-random placement in certain circumstances, such as when confirming the presence of a particular species or using mark-recapture methods, but otherwise we advocate the adoption of random survey locations and an emphasis on quality of data – as judged by the robustness and generality of conclusions that can be drawn – rather than quantity of data *per se*. This will be especially productive for study sites or study species which are poorly known, such as the bay cat, or for multi-species surveys, as a means of controlling for differential use of habitat features across species or between sexes within the same species.

## Supporting Information

File S1 Abstract (Malay translation).
(DOCX)
