# Supplementary material for: Assessing the Status of Wild Felids in a Highly-Disturbed Commercial Forest Reserve in Borneo and the Implications for Camera Trap Survey Design
Source: PLoS One. 2013 Nov 4;8(11):e77598. doi: 10.1371/journal.pone.0077598 (PMC3817192; doi:10.1371/journal.pone.0077598)
Supplement: File S1 — Abstract (Malay translation). (DOCX) [file pone.0077598.s001.docx]

**Penilaian status spesies kucing liar di Hutan Simpan Komersial yang teruk terganggu di Borneo dan implikasi rekabentuk kajian yang melibatkan kaedah perangkap-kamera**

**Abstrak**

Peningkatan dalam kajian yang melibatkan penggunaan perangkap-kamera telah memungkinkan julat taburan yang lebih luas telah diketahui terhadap banyak spesies kucing liar, tidak terkecuali di Borneo. Walau bagaimanapun, gambaran sebenar masih lagi tidak jelas tentang corak taburan kelimpahan spesies kucing liar di Asia Tenggara, terutama sekali di habitat yang teruk terganggu yang mana merangkumi kawasan yang luas. Satu faktor penting yang menjadi penghalang kepada penggunaan data dari kaedah perangkap-kamera secara lebih meluas ialah faktor perletakan perangkap-kamera di lokasi tak-rawak. Pemilihan lokasi perangkap-kamera yang tak-rawak akan berinteraksi dengan penggunaan ruang oleh haiwan secara tak-rawak dan ini menyebabkan salah tanggapan tentang kelimpahan relatif haiwan berdasarkan kepada maklumat kekerapan keboleh-kesanan haiwan. Perkara ini akan menjadi satu masalah sekiranya kajian gagal mengambil sampel yang mencukupi untuk merangkumi keseluruhan ciri-ciri habitat yang terdapat di sesuatu kawasan kajian. Dengan menggunakan rekod dari perangkap-kamera dan pemerhatian secara kebetulan daripada Hutan Simpan Kalabakan, Sabah, Borneo Malaysia, kami membuat penilaian yang bertujuan untuk menentukan kelimpahan relatif species kucing liar di hutan yang teruk terganggu, dan mengkaji penggunaan ruang oleh spesies kucing liar serta potensi kesan persampelan tak-rawak. Walaupun kawasan ini telah dibalak secara intensif sejak lebih dari tiga dekad yang lalu, didapati bahawa ia masih menampung kesemua spesies kucing liar yang terdapat di Borneo, termasuklah kucing merah, *Pradofelis badia*, iaitu satu spesies yang endemik di Borneo dan kurang diketahui. Dengan menggunakan perangkap-kamera yang diletak di lokasi yang rawak, empat daripada lima spesies kucing liar Borneo telah berjaya dikesan, yang mana spesies kucing liar ini menunjukkan perbezaan inter- dan intra-specifik dalam penggunaan ruang. Kami turut membandingkan data yang diperoleh dari kajian ini dengan set data yang besar iaitu >1,200 rekod berkaitan Felid dari kajian-kajian terdahulu yang melibatkan penggunaan perangkap-kamera. Kami dapati bahawa, kelimpahan relatif kucing merah, khususnya, mungkin lebih tinggi daripada apa yang dijangkakan sebelum ini akibat dari penggunaan data kajian dari perangkap-kamera yang diletak di lokasi secara tak-rawak. Kajian yang lebih lanjut terhadap spesies kucing merah menggunakan perangkap-kamera yang diletak di lokasi rawak adalah penting untuk menentukan status pemuliharaan Spesies kucing ini. Kami menyokong agar penyelidikan yang meletakkan perangkap-kamera di lokasi rawak perlu diperbanyakkan di masa hadapan demi untuk meningkatkan kebolehpercayaan dan deduksi umum bagi data yang diperoleh dari penyelidikan seperti ini.
